# Multiple light inputs to a simple clock circuit allow complex biological rhythms

**DOI:** 10.1111/j.1365-313X.2011.04489.x

**Published:** 2011-04

**Authors:** Carl Troein, Florence Corellou, Laura E Dixon, Gerben van Ooijen, John S O'Neill, François-Yves Bouget, Andrew J Millar

**Affiliations:** 1 School of Biological Sciences, University of Edinburgh and Centre for Systems Biology at EdinburghEdinburgh, EH9 3JD, UK; 2 University Pierre and Marie Curie Paris 06, Laboratoire d'Oceanographie Microbienne, Observatoire OceanologiqueF-66651 Banyuls sur Mer, France; 3 Centre National de la Recherche Scientifique, Laboratoire d'Oceanographie Microbienne, Observatoire OceanologiqueF-66651 Banyuls sur Mer, France

**Keywords:** circadian clock, *Ostreococcus tauri*, light inputs, photoperiod, model, phase response

## Abstract

Circadian clocks are biological timekeepers that allow living cells to time their activity in anticipation of predictable environmental changes. Detailed understanding of the circadian network of higher plants, such as *Arabidopsis thaliana*, is hampered by the high number of partially redundant genes. However, the picoeukaryotic alga *Ostreococcus tauri*, which was recently shown to possess a small number of non-redundant clock genes, presents an attractive alternative target for detailed modelling of circadian clocks in the green lineage. Based on extensive time-series data from *in vivo* reporter gene assays, we developed a model of the *Ostreococcus* clock as a feedback loop between the genes *TOC1* and *CCA1*. The model reproduces the dynamics of the transcriptional and translational reporters over a range of photoperiods. Surprisingly, the model is also able to predict the transient behaviour of the clock when the light conditions are altered. Despite the apparent simplicity of the clock circuit, it displays considerable complexity in its response to changing light conditions. Systematic screening of the effects of altered day length revealed a complex relationship between phase and photoperiod, which is also captured by the model. The complex light response is shown to stem from circadian gating of light-dependent mechanisms. This study provides insights into the contributions of light inputs to the *Ostreococcus* clock. The model suggests that a high number of light-dependent reactions are important for flexible timing in a circadian clock with only one feedback loop.

## Introduction

The majority of life on earth is greatly affected by the daily rhythms of sunlight, a fact that is reflected by the near-ubiquity of circadian clocks in living organisms. The oscillations of these daily clocks enable organisms to match their activities to the rhythmic environment by entraining to light and other cues. Circadian clocks in eukaryotes consist of multiple interlocked transcriptional feedback loops between sets of clock genes, in addition to environmental inputs that include light- and temperature-sensing pathways (Dunlap *et al.*, 2004). Plants are particularly dependent on sunlight, and their clocks link processes such as metabolism and growth to the rhythmic daylight to maximize fitness (Dodd *et al.*, 2005; Harmer, 2009; Graf *et al.*, 2010). Maintaining appropriately timed circadian rhythms is complicated by several environmental variables. First, temperature variations greatly affect biochemical reaction rates, requiring temperature compensation that is realised by balancing partially redundant clock components (Gould *et al.*, 2006; Akman *et al.*, 2008). Second, intrinsic noise and fluctuations in light intensity and quality require the oscillations to be robust against such perturbations, which favours networks with interlocked feedback loops (Cheng *et al.*, 2001; Smolen *et al.*, 2001). Finally, seasonal changes in day length require the clock components to oscillate with the appropriate amplitude and phase over a wide range of photoperiods (Roenneberg and Merrow, 2002; Eriksson and Millar, 2003). These factors suggest that even the simplest circadian clocks are intricate systems, with many components and interactions.

*Ostreococcus tauri*, described as the smallest free-living eukaryote (Courties *et al.*, 1994), is a marine unicellular alga with a minimal genome and a correspondingly simplified circadian clock. It possesses only a subset of the clock genes known in land plants such as *Arabidopsis thaliana*. Knowledge from Arabidopsis and the findings of Corellou *et al.* (2009) suggest the existence of a negative transcriptional feedback loop between *TOC1* and *CCA1* at the core of the *Ostreococcus* circadian clock. CCA1 protein, which is expressed in the night and early morning, is known to repress *TOC1* transcription by binding to the evening element motif in the *TOC1* promoter region (Harmer *et al.*, 2000; Corellou *et al.*, 2009). The TOC1 protein has a sharp expression peak in the evening, and appears to induce *CCA1* transcription thereafter.

Importantly, the *PSEUDO-RESPONSE REGULATOR* (*PRR*) and *LATE ELONGATED HYPOCOTYL* (*LHY*)/*CIRCADIAN CLOCK ASSOCIATED 1* (*CCA1*) families each have a single homolog (*TOC1* and *CCA1*) in the *Ostreococcus* genome (Corellou *et al.*, 2009), compared with five and eight, respectively, in Arabidopsis (Harmer, 2009). The *Ostreococcus* clock appears to have few components even in comparison with other chlorophyte algae, such as *Chlamydomonas reinhardtii* (Schulze *et al.*, 2010). It has been suggested that results from over-expression of *TOC1* might not be fully explained by a single negative feedback loop (Corellou *et al.*, 2009). However, it is worthwhile pursuing the single-loop hypothesis in order to define which results it can fully account for, and where its specific limitations lie. We have constructed a mathematical model using with the known components as a formal test of the single-loop hypothesis.

In addition to its reduced genomic complexity, *Ostreococcus* is easy to propagate and manipulate, and can readily be subjected to various and/or drug treatments in microplates within a single experiment. The expression of individual clock genes can be quantified *in vivo* through recently developed transcriptional and translational luciferase reporter lines (Corellou *et al.*, 2009), allowing differentiation of the effects of an experimental treatment between transcriptional and post-transcriptional processes. These factors make *Ostreococcus tauri* an ideal organism for a systems biology approach to understand the circadian clock in the green lineage.

**Figure 1:**
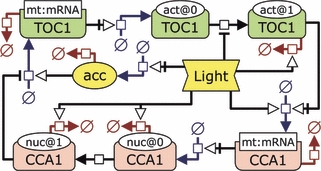
Scheme of the *Ostreococcus tauri* circadian clock model using the Systems Biology Graphical Notation (SBGN, http://www.sbgn.org/).The green and pink boxes represent the TOC1 and CCA1 mRNAs and proteins. Processes are drawn in blue for synthesis, red for degradation, and black for conversion or transport, and may be regulated by one or more components. Hollow arrows, bars and arrows with bars indicate positive and negative regulation and absolute requirement, respectively. TOC1 protein exists in two activation states, with light-regulated conversion from inactive (act@0) to active (act@1). Degradation of TOC1 protein is light-induced and only the active form is degraded. CCA1 protein exists in both the cytosol (nuc@0) and the nucleus (nuc@1), and is subject to light-induced degradation at the same rate in both compartments. ‘acc’ (yellow) is the ‘light accumulator’, which, by regulating *TOC1* transcription, links the overall gene expression levels to the amount of light received by the organism.

In this study, we have acquired a large set of experimental data in order to develop a mechanistic model of the *Ostreococcus* clock (Figure 1). The model reproduces the behaviour of the biological system over a wide range of light/dark input cycles, including the transient behaviours that follow a change of inputs. Detailed examination of the model's response to changes in photoperiod shows how a clock with only two genes at its core may generate a complex phase response. Finally, we show that, even under complicated light regimes, the model generates experimentally testable and useful predictions, providing directions for future research into this minimal biological clock.

## Results

**Figure 2:**
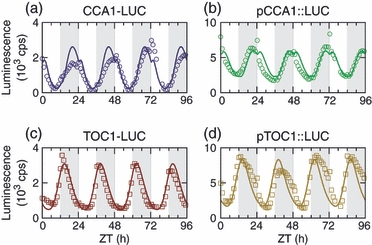
Dynamics of the core clock components.Measured data (points) are compared with the model (lines) over 12 h light/dark cycles (L:D 12:12, white/grey shading). The combined use of translational and transcriptional reporter lines (left and right, respectively) is useful in classifying features of the curves as caused by transcriptional or post-translational regulation. The data were re-scaled as described in Experimental procedures.

**Figure 3:**
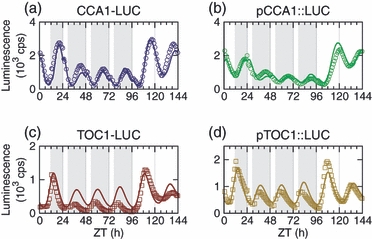
Transient effects of altered photoperiod.After entrainment and initial measurements in L:D 12:12, the photoperiod was shortened to 6 h by switching the lights off at subjective noon. After 3 days under L:D 6:18, the cells were moved into constant light. Measured data (points) are compared with the model (lines) for transcriptional and translations reporters for CCA1 and TOC1. The data were re-scaled as described in Experimental procedures.

**Figure 4:**
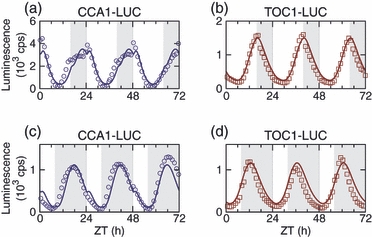
The model reproduces the clock dynamics under long and short photoperiods.Luminescence signals from long-day (L:D 16:8, top) and short-day (L:D 8:16, bottom) conditions were measured for CCA1–LUC and TOC1–LUC. The model output (lines) is in good agreement with the phase and general shape of the signals. The data were re-scaled as described in Experimental procedures.

**Figure 5:**
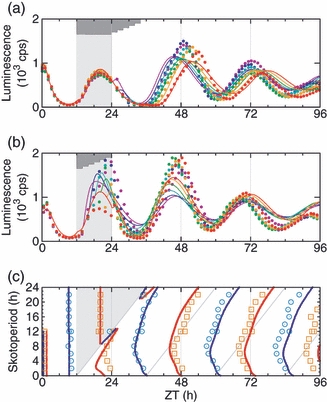
The model captures the complex observed phase response.After entrainment under L:D 12:12 conditions, the cells were exposed to a single dark period of variable length before entry into constant light.(a,b) Time courses for CCA1–LUC, comparing model (lines) with experimental data (points). (a) Late dawn; lights on at ZT0 to ZT10 in 2 h increments (purple, dark blue, light blue, green, yellow, red). (b) Early dawn; lights on at ZT14 to ZT24 in 2 h increments, coloured as in (a). Light grey indicates the expected 12 h night, dark grey indicates the range of actual nights.(c) Phase (*x* axis) versus photoperiod (*y* axis); the phases of peaks (red) and troughs (blue) are shown for the model (lines) and experimental data (hollow squares/circles).The data in (a) and (b) were re-scaled as described in Experimental procedures.

To construct a quantitative model of the *Ostreococcus* clock (Figure 1), we recorded a large number of luciferase luminescence time-series using both translational (TOC1–LUC and CCA1–LUC) and transcriptional (pTOC1::LUC and pCCA1::LUC) reporters (Figures 2–5, S1 and S2).

The timing and shape of TOC1 and CCA1 expression and the differences between transcriptional and translational reporters yield valuable information on the dynamics of the underlying biochemical processes that may be incorporated into the mathematical model. The modelling is based on the behaviour of the central clock genes under a wide array of light conditions. Special consideration has been given to the response of the clock to changes in the photoperiod, including the transient behaviour as the oscillator adapts to new conditions. Photoreceptors are not explicitly included in the model, nor are the pathways by which they affect the core clock genes. Although modelling of such details is clearly a future possibility, assuming sufficient knowledge is obtained about these details in *Ostreococcus*, we consider here only the effects that light, directly or indirectly, may have on biochemical reaction rates in the core clock. The ordinary differential equations and parameters of the model are described in the Experimental procedures and Appendix S1. The model equations have previously been used in a mathematical context (Akman *et al.*, 2010), but here we introduce and motivate the model from a biological viewpoint, and describe its workings and implications in detail.

### Observations under light/dark cycles

Before explaining the design and function of the model, we discuss the experimental observations over the course of a 12 h light/12 h dark cycle (L:D 12:12), as shown in Figure 2. We refer to the times at which the light is switched on and off as dawn and dusk, respectively. In the experiments, the TOC1–LUC level rose towards the end of the day and peaked sharply at dusk. Post-transcriptional processes are known to be important in the regulation of TOC1 (Djouani-Tahri *et al.*, 2010), and the data suggest that TOC1 protein is degraded more rapidly in the dark, in addition to a reduction or cessation of transcription caused by the increasing CCA1 level and the onset of darkness.

*CCA1* transcription was sustained throughout the night, with CCA1–LUC peaking 2–4 h before dawn, at which point TOC1–LUC had already fallen to a low level. At dawn, the onset of light triggered a sharp rise in CCA1–LUC for 1–2 h, leading to a second peak in the early morning. This morning peak was also seen for pCCA1::LUC (Figure 2). In addition, both CCA1–LUC and pCCA1::LUC show a transient dip at dusk, which delays the increase in expression at that time. Together, these observations suggest that the *CCA1* transcription rate is higher in the light, even though most *CCA1* transcription is timed to occur in the dark.

After the morning peak, the level of CCA1 fell throughout the day. About 4 h before dusk, the TOC1 reporter levels began to increase, with timing that was consistent with the picture of CCA1 as a repressor of *TOC1* transcription. The increase in *TOC1* transcription was followed by an increase in the levels of the CCA1 reporters an hour or two before dusk.

Changing the day length from 12 h affects the timing of clock gene expression, and reveals causal relationships between the clock genes and the light inputs. In addition, shape changes in the luminescence curves reveal specific details about other molecular processes, e.g. light dependence of degradation rates. In *Ostreococcus*, a notable difference between long and short photoperiods was the relative size of the CCA1–LUC morning peak (Figure 4). Entrainment under a longer photoperiod (L:D 16:8) resulted in higher expression in the morning peak, whereas a shorter photoperiod (L:D 8:16) reduced its level compared with results for the L:D 12:12 conditions shown in Figure 2.

### Model construction

Based on the data presented and cited above, we believe that TOC1 is the main regulator of *CCA1* transcription, either directly or indirectly, in *Ostreococcus*. Any model based on this assumption must account for the observed delay between the TOC1–LUC peak at dusk and the CCA1–LUC peak in the later part of the night. The same problem exists in Arabidopsis, although the timing of gene expression is different. In the first Arabidopsis clock model described by Locke *et al.* (2005a), transport of TOC1 from cytosol to nucleus created the necessary delay, but the profile of TOC1 mRNA and protein was inconsistent with experiments. Locke *et al.* (2005b) solved this by introducing a hypothetical transcription factor, X, located between TOC1 and *CCA1* in the loop. However, they also mentioned another possibility: ‘either the active form of TOC1 is present at a far lower concentration than bulk TOC1 protein, perhaps in a complex, or … an additional, TOC1-dependent component is the direct activator of *LHY* and *CCA1*’.

We do not follow the Arabidopsis clock model in introducing an unknown component to delay the signal from TOC1 to *CCA1.* Instead, we explore the alternative (and no less parsimonious) hypothesis that TOC1 can exist in two states: inactive and active. Under this hypothesis, TOC1 is synthesized in the inactive form, and is slowly activated in the dark to give a broad peak of active TOC1, and thus *CCA1* transcription, in the later half of the night. We cannot experimentally observe the hypothetical activation of TOC1, because only total TOC1–LUC is measured. However, for the model to be consistent with the experimental profile of total TOC1, it is necessary for the active form of TOC1 to be present at a significantly lower level than the inactive form. Consequently, the model may be very similar, in terms of its equations, to one in which ‘active TOC1’ is replaced by a distinct, post-transcriptionally regulated transcription factor. For example, the majority of inactive TOC1 could be degraded and never activated. The detail of this mechanism is not crucial, however. In the model presented here, all TOC1 passes from the inactive to the active form (see below).

To explain the CCA1 morning peak and its transient nature, the model assumes that the active form of TOC1 is a stronger activator of *CCA1* transcription, but also more rapidly degraded, in the light than in the dark. Any active TOC1 remaining at dawn causes a short-lived increase in *CCA1* transcription. In addition, the model requires a higher CCA1 protein degradation rate in the light than in the dark, as indicated by the differences between pCCA1::LUC and CCA1–LUC profiles in the experiments (for example, Figure 2). The model is able to reproduce qualitatively the correlation between photoperiod and the *CCA1* morning peak, solely through transcriptional regulation of *CCA1* by TOC1 protein. This is possible because, in the model, the length of the night is important in determining the amount of active TOC1 that remains to activate *CCA1* at dawn. The mechanism in the model represents a hypothesis that is quantitatively consistent with the data. It was identified by our computational approach and can be understood as described below.

As originally conceived, the model included degradation of both the active and inactive forms of TOC1 protein, as well as light-dependent conversion between the two forms. The process of fitting model parameters to match the experimental data eliminated some of these reactions because they were redundant, resulting in a model in which TOC1 must be activated before it can be degraded. Consequently, the rate at which a pool of TOC1 is degraded in the model depends on what fraction is in the active form. According to the best fitted parameter values (Table S1), the rate of TOC1 activation, which is higher in the dark than in the light, is a bottleneck to TOC1 degradation. Thus, total TOC1 in the model was degraded more quickly in the dark, even though the active form was more stable in the dark. The model's dynamic behaviour, using this mechanism, was consistent with the data on total TOC1 and on the activation of *CCA1* under various light:dark cycles.

Experiments showed that, if the day is shortened by an early dusk, the pTOC1::LUC reporter level nevertheless increased at around the predicted time of dusk in the L:D 12:12 cycle, but to a lower level than it otherwise would (Figure S1). The level of *TOC1* expression was roughly proportional to the photoperiod (Figure S3), despite the absence of light. Such a response suggests that the processes that control the *TOC1* transcription rate effectively retain a memory of the amount of light received by the cell. We cannot identify the positive regulators of *TOC1* transcription, but photoreceptor signalling and/or metabolic state might be involved. To describe this process in the model, we have placed *TOC1* transcription under the positive control of a ‘light accumulator’ component (‘acc‘ in Figure 1), whose value slowly approaches 1 in constant light and 0 in constant darkness.

The equations in our model include diffusion of CCA1 between the cytosol and the nucleus, but this diffusion rate is maximized in the best fitted parameter set, and thus we may consider cytosolic and nucleic CCA1 to be in rapid dynamic equilibrium.

### Modelling transient behaviour

The relative simplicity of the *Ostreococcus* system, coupled with high experimental reproducibility and time resolution, has enabled us to fit the model directly against many measured time courses. This differs from the approach taken for Arabidopsis, where development of successive clock models was aided by reduction of noisy biological data into more robust measures, such as the period and phase of oscillations. Fitting the model using a least-squares-like cost function (see Experimental procedures) gives weight to all features of the time courses, including phase and shape, at the expense of classical circadian observables, such as period in constant light.

Plant clock models have hitherto primarily considered limit cycle oscillations, which represent the behaviour after long entrainment periods. In contrast, a majority of the experiments used for fitting the *Ostreococcus* clock model contain a change in the photoperiod, transfer of the cells into constant light, or both. Figure 3 shows that the model is able to track the levels of the measured clock components across these changes in the light conditions. Such tracking requires the model to reproduce the amplitude, period, phase and general shape of expression as the conditions change, which is more challenging than reproducing only the phase and period of oscillations. In general, the simulations and experiments show a high level of agreement. The differences between model simulations and data are quantified by a local sum square error, normalized to the error value for a straight line through the data, such that an error of 1 indicates a model output as bad as a straight line. The best parameter set has an error of 0.39. Thus the model can explain over 60% of the variation in measured luminescence levels when averaged across all 144 experiments used to fit the model (see Experimental procedures).

### The effects of photoperiod

The relationship between photoperiod and phase holds information about the overall function of the clock and the contributions of the individual clock components. A change in photoperiod causes a phase response in the clock components, which can be quantified by the shift in timing of the peaks and troughs compared to the preceding entrainment period. We have performed a series of experiments in which the photoperiod was changed from 12 h to any length between 2 and 22 h, in increments of 2 h, by altering the time of dusk or dawn. In one version of the experiments, the photoperiod was only altered for a single day before release into constant light (LL), in order to reveal when and how the phase of the clock is sensitive to transitions between light and dark. In other experiments, the cells were exposed to the new photoperiod for 3 days, which is sufficient for the *Ostreococcus* clock to reach stable entrainment, revealing both the transient effects of photoperiod entrainment and the effects of photoperiod on phase in LL.

Figure 5 shows the effects on CCA1–LUC when cells were transferred from L:D 12:12 into constant light, and when the final night was either extended or shortened. Progressively later dawns caused greater phase delays. However, the delays were less than they would be if an extended night caused a complete resetting of the clock. Similarly, an earlier dawn caused a phase advance. The size of the phase advance is greatest when dawn is 6 h earlier than in the preceding L:D 12:12 cycle. Smaller phase advances were observed in response to even earlier dawns. These data show that the resulting clock phase is not locked directly to either dawn or dusk, but instead shows a complex response to the perturbation. This flexibility in circadian phase was also observed in the results for 3 days of altered photoperiod (Figure S1).

The model predicts the first peak in LL to occur earlier than it did experimentally, and over-estimates the period of the oscillations, probably due to the small amount of LL data used in the model fitting (see Table S2). However, it is clearly able to reproduce the overall experimental phase response. Such a complex response to the light input signal has been observed in other organisms (for example, Edwards *et al.*, 2010), but it was surprising to see in the model. Experience from other clock models (e.g. Locke *et al.*, 2006; Akman *et al.*, 2008) indicates that interlocked feedback loops, which this model lacks, are required for such non-trivial relationships between clock phase and light input.

Closer comparison between photoperiods (Figures 2 and 4) sheds light on how both dawn and dusk are important in setting the phase of the oscillator in the present model. Under short days, the dark-induced transformation of TOC1 protein into its active form causes the levels of CCA1 mRNA and protein to peak earlier, whereas the longer night results in less TOC1 at dawn and consequently a smaller peak of CCA1 in the morning, which in turn causes an earlier CCA1 trough that enables an earlier peak in TOC1 mRNA and protein. Under long days, the additional *CCA1* transcription before dusk leads to higher CCA1 protein levels at dawn, but has little impact on the CCA1 trough. However, the longer days allow TOC1 protein to keep rising, leading to later and higher peaks of both TOC1 and CCA1 protein. The varying contributions of these mechanisms offer an explanation as to how the clock model can avoid locking its oscillations to either dawn or dusk. Circadian gating of input signals is sometimes seen as the task of an external ‘*zeitnehmer*‘comprising additional molecular components (Roenneberg and Merrow, 1999; McWatters *et al.*, 2000), but here gating of the light input signal is a property of the oscillator mechanism. Our results indicate that a single feedback loop may indeed be capable of generating the complex phase response observed in *Ostreococcus* (e.g. Figure 5).

### Contributions of light inputs

The biological system, like the model, may rely on circadian gating of the input signal into various parts of the loop. It is therefore of interest to explore the roles that the individual light inputs play in the model, so that understanding of the model can direct future experimental work. Table S3 summarizes the effects that light has on the behaviour of the model at various time points. However, light enters the model in five different places, and it is not easy to determine what each light input contributes to the function of the system as a whole. This is an issue that is largely separate from the question of what photoreceptor pathways are involved in controlling the biological clock, and can be addressed though simulations.

**Figure 6:**
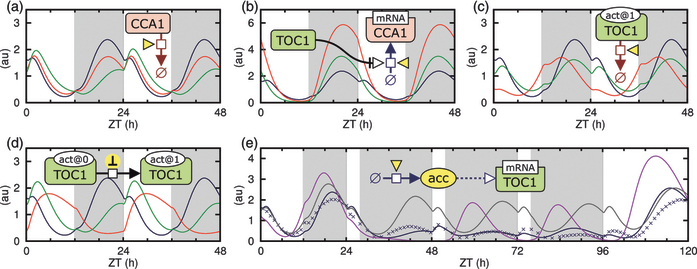
All five light inputs are important for the function of the model.In each panel, the dynamics of CCA1–LUC are compared between the ‘wild-type’ model (blue lines) and mutated models in which one light input is disabled.(a–d) L:D 12:12 conditions; the rate constant governed by the examined light input is set equal to the value it takes in the light (red) or dark (green) in the wild-type model. (a) Degradation rate of CCA1. (b) TOC1-mediated transcription of *CCA1*. (c) Degradation rate of active TOC1. (d) TOC1 protein activation rate.(e) The function of the light accumulator (acc) is demonstrated across two photoperiod changes, with *TOC1* transcription either made independent of the light by setting acc to 1 (grey), or modulated directly by the light, bypassing acc (purple). Experimental data (blue crosses) are included for comparison, and have been re-scaled as described in Experimental procedures.

The model has four light-regulated reaction rates, each with separate rate constants for light and dark. We have disabled one light input at a time by setting its rate constants to identical values to mimic the effects of switching a hypothetical regulator on or off. A slightly different approach was used for regulation of *TOC1* transcription through the ‘light accumulator’, because the relevant model parameter represents a time scale rather than a rate constant (Figure 6). From this analysis, we are able to deduce the role of each light input in the model.

#### CCA1 degradation

Stabilization of the CCA1 protein in the dark ensures that, after dusk, the level of CCA1 can rapidly increase and switch off *TOC1* transcription (Figure 6a).

#### CCA1 transcription

The characteristic CCA1–LUC signal profile can only be reproduced if *CCA1* transcription is more strongly light-activated than the degradation of CCA1 protein. This light-activated transcription appears to facilitate entrainment by boosting the repression of *TOC1* transcription by CCA1 protein at a well-defined phase, namely dawn (Figure 6b).

#### TOC1 degradation

A low TOC1 protein degradation rate in the dark is necessary if the TOC1 expressed in the evening is to drive transcription of *CCA1* during the night and morning. Light-induced degradation of TOC1 protein is therefore required for a strong separation between the TOC1-LUC trough and peak levels (Figure 6c).

#### TOC1 activation

Dark-induced conversion of TOC1 protein into the active form is central to the function of the system, as can be seen from the profound impact that removing this light input has on phase (Figure 6d).

#### TOC1 transcription

If *TOC1* transcription were independent of light, the observed coupling between photoperiod and signal amplitude would be lost, but direct regulation by the light can also be ruled out because it would cause complete resetting of the clock after long nights. The existence of a proposed ‘light accumulator’ that regulates *TOC1* transcription fits with the observed changes in both amplitude and phase (Figure 6e).

All five light inputs contribute to the model's dynamics in distinct ways. This finding suggests that the dynamics are highly dependent on the parameters, with considerable flexibility in adjusting the model's rhythmic behaviour. At the general level, such flexibility may explain why the model is able to reproduce the complex behaviour of the *Ostreococcus* clock.

### Over-expression mutants

**Figure 7:**
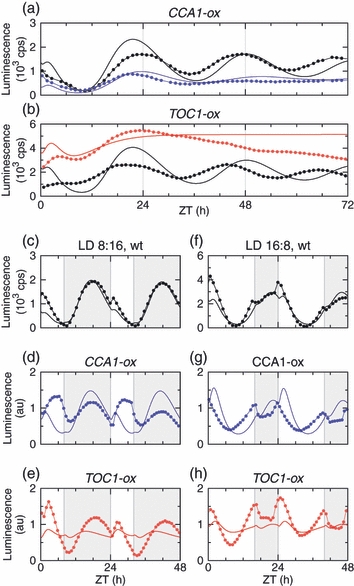
The effects of *CCA1* and *TOC1* over-expression are captured by the model.(a, b) The model reproduces the change in CCA1–LUC period and expression level between wild-type (black) and the over-expression mutants *CCA1-ox* (blue) and *TOC1-ox* (red). Data are shown as points connected by lines; model predictions are shown as solid lines. Within either panel, the simulated data were scaled by the same factor for wild-type and mutant.(c, d) CCA1–LUC expression in the wild-type under light/dark cycles with various photoperiods. The scaling of the simulated data is the same in (c) and (d).(e–h) Predictions for the CCA1–LUC signal in the mutants under light/dark cycles, using the same levels of *CCA1* and *TOC1* over-expression as in (a) and (b). The scaling of the data in (e–h) is arbitrary.

A major advantage of the clock model is that it can generate experimentally testable hypotheses. Depending on the outcome, these experiments can validate the model, identify results that merit further investigation, or both. The over-expression mutant lines for *CCA1* and *TOC1* in the CCA1–LUC reporter background (Corellou *et al.*, 2009) provide a powerful means to test the predictive power of the model. Over-expression of *CCA1* or *TOC1* was introduced into the model equations as described in Appendix S1. For each of the two genes, the level of over-expression was adjusted to make the model fit the change in luminescence level between wild-type and mutants under constant light (Figure 7). The changes in expression level were accompanied by predicted changes in the model's period and damping rate, which were in good agreement with the experiments.

Constitutive over-expression of *CCA1* in *CCA1-ox*/CCA1-LUC is predicted to correspond to four times the normal trough level (or half the peak level) of *CCA1* expression in L:D 12:12. The over-expression of *TOC1* in *TOC1-ox*/CCA1-LUC is 14 times the trough level (half the peak level). These estimates are in line with the RT-PCR data reported by Corellou *et al.* (2009).

Figure 7 also shows predictions for CCA1–LUC expression profiles under light/dark cycles with long or short days. The model predicts that, in the *CCA1-ox* background, the phase of the CCA1–LUC trough under long days is advanced compared with wild-type, and this is verified by the experimental data. Another experimentally verified prediction is the increased CCA1–LUC morning peak following *TOC1* over-expression, particularly under the short photoperiod. Although the model predictions are not quantitatively accurate, the qualitative validity demonstrates that the model is based on sound biological assumptions: no additional mechanisms are required to account for these data.

### Skeleton photoperiod

The response to new regimes of light input is highly relevant to test the *Ostreococcus* clock model, particularly with respect to circadian gating. Circadian clocks in animals and plants can be entrained to a skeleton photoperiod, where light is supplied near dusk and dawn but the remaining part of the subjective day is dark (Pittendrigh and Minis, 1964; Hillman, 1972). Because of the low total amount of light that the cells receive in such experiments, we have also considered the response of *Ostreococcus* to a skeleton skotoperiod where light is added in the middle of the subjective night.

**Figure 8:**
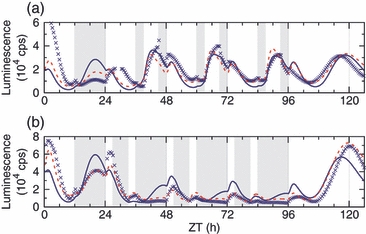
The response to skeleton light conditions depends on the light intensity.The predictive power of the model was tested by replacing the middle 6 h of the night (a) or day (b) in L:D 12:12 with light (a) or darkness (b), for three cycles, followed by transfer to constant light. This experiment used a lower light intensity than previous experiments (see Experimental procedures). The measured response of CCA1–LUC (blue crosses) is similar to the model prediction for normal light intensity (solid blue lines), but is better matched by the prediction for low (30%) light intensity (dashed red lines).

We have tested the clock model against experiments under both types of skeleton photoperiod (Figures 8 and S3). Cells were transferred from L:D 12:12 to the test cycle, then to LL. As expected, the overall shape of the luminescence response was correctly predicted by the model under both conditions. However, the model exhibited noticeable deviations from the experimental observations for the skeleton photoperiod (Figure 8b), especially for the timing of CCA1–LUC expression after release into constant light. The skeleton experiments were performed under a lower light intensity than the experiments used for model fitting, and the limited duration of light appears to be insufficient for oscillations to be sustained to the extent that the model predicts. Accordingly, we adjusted the model parameter that corresponds to the light intensity, reducing the effect of light on the ‘light accumulator’ by 70% to match the decreased amplitude of CCA1–LUC. Figure 8 shows that this adjustment is sufficient to make the model correctly predict the peak time for CCA1–LUC in constant light following the skeleton photoperiod. The predictions from the modified model also match the experimental data for the morning peak in CCA1–LUC under the complete (non-skeleton) light/dark cycles that preceded the transfer to skeleton photoperiods (Figure S4), further supporting the parameter adjustment. Low light was predicted to have little effect on clock gene expression under skeleton skotoperiod cycles, consistent with the data (Figure 8a). In particular, the phase in LL was predicted and observed to agree with data from complete photoperiods of the higher light intensity (Figure 5a). Together, these findings indicate that, under low light, the system is sensitive to the total amount of light rather than the light intensity itself.

## Discussion

We have developed a mathematical model of the *Ostreococcus tauri* circadian clock as a feedback loop between *TOC1* and *CCA1*, guided by experimental observations under many light conditions. The model reproduces the dynamics of transcriptional and translational luciferase reporters for both genes, over a range of photoperiods, and is even able to predict the transient response of the clock when the light conditions are altered. This detailed model fitting was made possible by the high reproducibility of the luminescence data from *Ostreococcus* cell cultures. The model's ability to match these data, and to predict the cell's rhythmic responses to genetic and environmental perturbations, supports the validity of the proposed clock gene circuit. Systematic examination of the model's behaviour helped us to explain and predict how the *Ostreococcus* circadian clock can produce complex light responses, despite having a minimal number of genes compared with higher plants.

Some biochemical processes underlying the model remain to be elucidated. In particular, the mechanism by which TOC1 protein regulates *CCA1* transcription is not known in any species. Recent results suggest the possibility of direct DNA binding via the CCT domain of TOC1 (Tiwari *et al.*, 2010). The Arabidopsis clock models described by Locke *et al.* (2005b, 2006) included the hypothetical component X as the delaying link between TOC1 expression and later *CCA1* transcription. The *Ostreococcus* model presented here eliminates X, like the latest Arabidopsis clock model (Pokhilko *et al.*, 2010). Instead, these models include an activated form of TOC1, which, in our model, is light-dependent. As previously noted (Locke *et al.*, 2005b), these are two interpretations of a fundamentally similar, TOC1-dependent activation of *CCA1* expression.

Protein phosphorylation is important in regulating circadian clocks (Gallego and Virshup, 2007), and provides a possible mechanism for both the activation and degradation of TOC1. In Arabidopsis, there is a complex interplay between the PRR and TOC1 proteins, with important consequences for the phosphorylation, interactions, degradation and nuclear localization of TOC1 (Fujiwara *et al.*, 2008; Wang *et al.*, 2010). Similar mechanisms may operate in *Ostreococcus*, where TOC1 is the only homologue of the higher-plant PRRs. However, in contrast to these proteins, *Ostreococcus* TOC1 possesses a phosphate-receiving aspartate residue in its receiver domain (Corellou *et al.*, 2009). If TOC1 is a functional response regulator, then time- or light-dependent transfer of phosphate to this residue may affect TOC1 function.

*TOC1* transcription showed an unexpected light response that was required for the model to match the data. The total light fluence received over the past cycle activates *TOC1* transcription via the model's ‘light accumulator’. This behaviour was directly indicated by the data for various photoperiods (Figure S6) and skeleton cycles (Figures 8 and S4), but is unusual among clock models. Rapid light responses were sufficient to model calcium levels and microarray data in Arabidopsis (Dalchau *et al.*, 2010), as well as detailed profiles of clock gene expression (Edwards *et al.*, 2010; Pokhilko *et al.*, 2010). Either a regulatory photoreceptor or a metabolic product of photosynthesis could be responsible for the integration of light signals. Commitment to cell division also depends on the total fluence received by *Ostreococcus* cells, whereas the timing of cell division is gated by the clock and locked to dawn (Moulager *et al.*, 2007). Translation of the key G_1_ protein cyclin A, which is required for S-phase entry, depends on a similar light accumulator (Moulager *et al.*, 2010). Thus a common mechanism might link *TOC1* transcription and cell-cycle progression.

In general, the clock model predicted CCA1 dynamics better than TOC1 dynamics. The obvious interpretation is that the regulation of CCA1 is more accurately described in the model, but this is not necessarily true given the feedback nature of the system. Our experimental results tested the limits of detailed predictions. For example, CCA1–LUC expression appeared almost saturated in simulations of the *TOC1-ox* line (Figure 7), suggesting that *CCA1* transcription in the model is too sensitive to small changes in the TOC1 protein level. This indicates that only a quantitative adjustment is required to a future model. In contrast, the model cannot account for an additional peak of TOC1–LUC expression in response to an early dawn (Figure S2), suggesting that another regulator gains importance under these conditions. The model also highlights evidence that CCA1 is not controlled solely by TOC1. Under skeleton photoperiods, CCA1–LUC responded differently to light pulses at dawn and dusk (Figure 8). The model and data indicate that TOC1 is at a low level at both times, suggesting a similar response to light. These results suggest that *Ostreococcus* contains an additional component that regulates CCA1 in a time-dependent fashion. Under normal conditions, its impact may be small, because our model lacking this component accurately matched data for complete light/dark cycles.

A common theme in circadian systems is the presence of multiple, interlocking feedback loops, suggesting that such complexity typically improves fitness. Day length changes with the seasons, and multiple feedback loops are necessary if some clock outputs are to be entrained to dawn and others to dusk (Pittendrigh and Daan, 1976; Rand *et al.*, 2004; Troein *et al.*, 2009). It is natural, therefore, to expect that a clock with a single feedback loop would only be capable of following dawn or dusk, as observed for the Arabidopsis clock models (Edwards *et al.*, 2010). However, our model demonstrates that even a single loop can produce complex phase relationships, provided there are multiple paths by which light enters the system. We have shown that the malleability of the model's response to light stems from circadian gating of the various light-dependent reactions, which have distinct effects on the system's behaviour. At the most general level, we conclude that a high number of light inputs may be required for flexible biological timing in any circadian clock with a simple circuit or few components.

## Experimental Procedures

Data for Figures 2–5, 7, S1 and S2, and for model fitting (Table S2), were obtained as described by Corellou *et al.* (2009). Transgenic lines of *Ostreococcus tauri* strain OTTH0595 were entrained and recorded under white light at 16 μmol m^−2^ sec^−1^. In the phase shifting (early/late dawn/dusk) experiments, the constant light was provided at 8–16 μmol m^−2^ sec^−1^.

### Cell culturing

The transgenic *Ostreococcus tauri* lines have been described previously (Corellou *et al.*, 2009). Cells were cultured in Keller media (Km) (K1630; Sigma, http://www.sigmaaldrich.com/) supplemented with artificial sea water (Instant Ocean; Aquarium Systems Inc., http://www.instantocean.com/) (Km) under 12:12 h light/dark cycles in soft white fluorescent light filtered with one layer of 724 Ocean Blue (Lee Filters, http://www.leefilters.com/) (17.5 μmol m^−2^ sec^−1^).

### Luminescence recording

Cultures were transferred to 96-well microplates (Lumitrac; Greiner Bio-one Ltd, http://www.greinerbioone.com/) at a density of approximately 15 × 10^6^ cells ml^−1^, and entrained for 7 days. One day prior to recording, 150 μl of medium was replaced with 150 μl Km containing 333 μm d-luciferin (Km+) (*Biosynth AG*). Bioluminescence recordings were performed on a TopCount (Packard/PerkinElmer) under red + blue LED light (3–12 μmol m^−2^ sec^−1^, limited by increasing evaporation at higher fluence rates) (NIPHT Ltd) controlled to the required photoperiod. For skeleton photoperiods, cells were entrained under L:D 12:12 before being moved to recording.

### Model construction

The basis for our model is time-course data from a large number of experiments and replicates, in which cultures of the transcriptional and translational reporter lines were exposed to light/dark (L:D) cycles of various lengths, and to transitions between various L:D cycles and/or into constant light (LL). Assuming stochastic effects to be small in these cell population data, we used ordinary differential equations, with variables for the mRNA and protein concentrations of TOC1 and CCA1. As described in Results, the model postulates inactive/active forms of TOC1 and cytosolic/nucleic CCA1. The full model (see Appendix S1) explicitly includes the reporter constructs (luciferase fusion proteins or luciferase mRNA and protein). The core equations are:


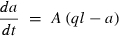



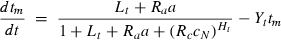



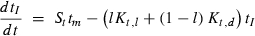



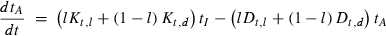



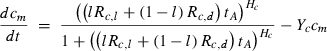



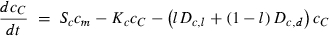



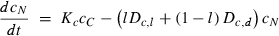


where *a* is the light accumulator, *l* is the light input signal (0 or 1), *q* is the light intensity (1 or 0.3; see text), *t* is TOC1 (with subscripts *m* for mRNA, *I* for inactive and *A* for active) and *c* is CCA1 (with subscripts *m* for mRNA, *C* for cytosolic and *N* for nucleic). Upper-case letters indicate parameters, some with subscript *t*/*c* for TOC1/CCA1, some with *d*/*l* for dark/light. *A* indicates the light accumulator change rate, *L* indicates light accumulator-independent transcription, *R* indicates the binding affinity, *H* indicates the Hill coefficient, *Y* indicates the mRNA degradation rate, *S* indicates the synthesis rate, *K* indicates the conversion/transport rate and *D* indicates the protein decay rate. The transcription rates are based on activators and repressors competing for binding as modelled by Shea and Ackers (1985). Degradation, conversion and transport all follow simple mass-action kinetics.

### Parameter optimization

The model's light inputs are the result of testing many possibilities. Modulation by light was only retained where it was required by the best parameter sets. To fit parameters, we repeatedly applied both a genetic algorithm and local optimizers, starting from the best of many initially random parameter guesses. The cost function was defined as the root mean square (RMS) of a local least-squares error measure across 144 experiments (Table S2). For each experiment, the system was simulated using a particular light input signal, starting from variable values that were themselves parameters to be fitted. Scaling between experimental and simulated data was calculated for each sample point individually, using a weighted 72 h time window, and the sum square error was calculated from this local scaling (see Appendix S1 for details). This cost function ensures that long-term trends in the data have little impact. Before the RMS averaging, the error for each experiment was normalized against the would-be error of a flat line, so any useful model would have a total cost well below 1. Our final best parameter set (Table S1) has a cost of 0.391.

The period of CCA1–LUC in constant light is somewhat too long in the model (25.4 h, compared with 24 h in experiments). This is partly explained by the long period of the TOC1–LUC reporter. Also, there was no explicit fitting of the period, and the experiments only include short periods of LL.

### Data scaling

The experimental data in the figures were re-scaled to counter exponentially decaying luminescence signal levels (see Appendix S2 and Figure S5). The values were multiplied by exp(*t*/*θ*), where 100 h ≤ *θ*≤ 240 h. The simulated data were scaled using an arbitrary factor to match the observed level in each plot.

## Note added in proof

During the preparation of this paper, a simple mathematical formulation has been developed to describe the Ostreococcus clock system based on RNA timeseries data (Thommen *et al.*, 2010; Morant *et al.*, 2010).

## SUPPORTING INFORMATION

**Figure S1.** Effect of perturbation of the time of dusk.

**Figure S2.** Effect of perturbation of the time of a single dawn for TOC1–LUC.

**Figure S3.** Levels of TOC1–LUC under skeleton light/dark cycles.

**Figure S4.** Effect of transitions between long and short days.

**Figure S5.** Signal decay and rhythmic damping have different time scales.

**Figure S6.** Effect of light on *TOC1* expression.

**Table S1.** Model parameters.

**Table S2.** The data sets used to fit the model parameters.

**Table S3.** Effects of light on the behaviour of the model at various time points.

**Appendix S1.** Modelling details.

**Appendix S2.** Damping oscillations in LL.

Please note: As a service to our authors and readers, this journal provides supporting information supplied by the authors. Such materials are peer-reviewed and may be re-organized for online delivery, but are not copy-edited or typeset. Technical support issues arising from supporting information (other than missing files) should be addressed to the authors.
