# Supplementary figures and images for: Multiple light inputs to a simple clock circuit allow complex biological rhythms
Source: Plant J. 2011 Apr;66(2):375–85. doi: 10.1111/j.1365-313X.2011.04489.x (PMC3130137; doi:10.1111/j.1365-313X.2011.04489.x)

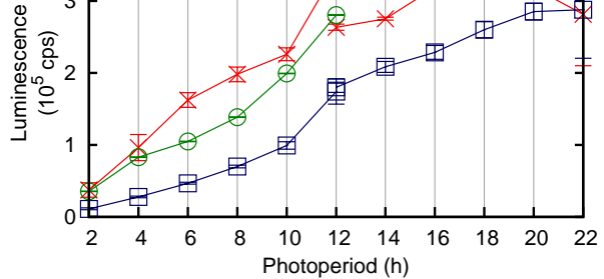

Supplement: Supplementary file 6 [file tpj0066-0375-SD6.pdf]
