# Supplementary material for: Multiple light inputs to a simple clock circuit allow complex biological rhythms
Source: Plant J. 2011 Apr;66(2):375–85. doi: 10.1111/j.1365-313X.2011.04489.x (PMC3130137; doi:10.1111/j.1365-313X.2011.04489.x)
Supplement: Supplementary file 7 [file tpj0066-0375-SD7.doc]

**Figure S1.** Perturbation of the time of dusk.

The transient effects of altering the time of dusk is reproduced by the model over a wide range of photoperiods, for both CCA1 and TOC1. After entrainment in LD 12:12 conditions, the photoperiod was changed by turning the lights on or off at new times, for three days, followed by entry into constant light. (a-b) Time courses for CCA1-LUC, comparing model (lines) with experimental data (points). (a) Early dusk (short days); lights off at ZT 2 (purple) to ZT 12 (red) in 2-hour increments. (b) Late dusk (long days); lights off at ZT 12 (purple) to ZT 22 (red). (c) Phase (*x* axis) versus photoperiod (*y* axis) for CCA1-LUC in early/late dusk. The phases of peaks (red) and troughs (blue) are shown for the model (lines) and experimental data (hollow squares/circles). (d-l) Data and experiments for (d-f) TOC1-LUC, (g-i) pCCA1::LUC and (j-l) pTOC1::LUC, presented in the same way as CCA1-LUC.

**Figure S2.** Perturbation of the time of a single dawn for TOC1-LUC.

The transients effects on TOC1-LUC of perturbing the time of dawn, presented as in Figure 5. The apparent discrepancy between the excellent fit for CCA1-LUC and the (initially) worse fit for TOC1-LUC can be explained: If dawn is advanced so that the light is switched on at some point during the later half of the night, the model predicts that TOC1 in its active form will transiently boost CCA1 transcription, causing a phase advance of nearly the same magnitude as the advance of dawn. If dawn is shifted to even earlier in the night, it is instead predicted to cause a net stabilisation of TOC1. However, the experiments show that the TOC1 level temporarily increases when the light is switched on, then continues to drop, whereas the model claims that the level will just drop more slowly after dawn. In both cases, a greater advance of dawn results in a shallower TOC1 trough in the morning. This causes the CCA1 trough level to rise, which in turn makes TOC1 to rise more slowly, leading to a lower, delayed peak.

**Figure S3.** TOC1-LUC in skeleton light/dark cycles.

The prediction of TOC1-LUC in is not as good as that of CCA1-LUC (Figure 8). Experimental data (red crosses) are compared with the model in its original form (solid red lines) and modified for dimmer light (dashed green lines), in the same light conditions as in Figure 8. The TOC1 level is sensitive to the total amount of light, if it falls below some threshold as seen for the skeleton photoperiod (lower panel). The much higher baseline incorrectly predicted by the model is only possible if there is strong nonlinearity in the transcriptional activation of *CCA1* by TOC1, which implies that the Hill coefficient for that reaction is overestimated in the model.

**Figure S4.** Transitions between long and short days.

CCA1-LUC measurements (blue crosses) are compared with mode simulations in two different patterns of long (LgD, LD 16:8) and short (ShD, LD 8:16) days. Simulations with the original light level (blue solid lines) and the lower light level from Figure 8 (red dashes) are similar but differ in the size of the CCA1 morning peak. Comparison with the data suggests that the light level in this experiment was higher than in the skeleton photoperiod experiment but lower than in the original model. The cells were entrained the light conditions of the first two days of measurement.

**Figure S5.** Signal decay and rhythmic damping have different time scales.

(a) Two independent time courses of CCA1-LUC data in constant light, after release from LD 12:12. The drop in the signal level is relatively rapid in these examples, compared to many other experiments. (b) The same data on a log scale, with lines to indicate the exponential decay of the mean signal levels. The time constant of the decay differs between the data sets (53 and 65.5 hours). (c) The data after subtraction of the exponential decay, showing the damping in rhythmic amplitude. (d) The same data multiplied with a growing exponential (time constant 18 hours) to negate damping. The relatively rapid damping of the circadian rhythm is identical between replicates, even though the slower signal decay is not.

**Figure S6.** The effect of light on *TOC1* expression level.

The mean luminescence signal from *TOC1* reporters is plotted, as a function of the total amount of light, from the data shown in Figure S1 and similar, unpublished data. Data are from the third day of altered photoperiod: pTOC1::LUC in altered dusk (red/crosses), TOC1-LUC in altered dusk (blue/squares) and TOC1-LUC in altered dawn (green/circles). Error bars represent standard deviation of the mean, n=4 cultures. *TOC1* expression level increases linearly with the amount of light for photoperiods of up to 10 hours.
