# Supplementary material for: Multiple light inputs to a simple clock circuit allow complex biological rhythms
Source: Plant J. 2011 Apr;66(2):375–85. doi: 10.1111/j.1365-313X.2011.04489.x (PMC3130137; doi:10.1111/j.1365-313X.2011.04489.x)
Supplement: Supplementary file 8 [file tpj0066-0375-SD8.pdf]

Table S1. Model parameters.
The best fit between the model and the experimental data was found with this parameter set. The parameters were optimised by application of a genetic algorithm followed by a local search. Concentrations in the model are in arbitrary units, presented in the "Units" column as "[]", whereas time is measured in hours.

Parameter	Value	Units	Description
A	0.08201	h^{-1}	Light accumulator decay/saturation timescale
R_t	1.0871	$[]^{-1}$	Strength of repression of *TOC1* by CCA1
H_t	2.0781	-	Cooperativity for repression of *TOC1* by CCA1
L_t	0.0001	-	TOC1 transcription in darkness
R_a	0.2311	$[]^{-1}$	Strength of *TOC1* transcription activation by *acc*
Y_t	0.2921	h^{-1}	*TOC1* mRNA degradation rate
S_t	0.7700	[]	*TOC1* transcription rate scale factor
$K_{t,l}$	0.1365	h^{-1}	Rate of TOC1 conversion to active form, light
$K_{t,d}$	0.3266	h^{-1}	Rate of TOC1 conversion to active form, dark
$D_{t,l}$	0.4616	h^{-1}	TOC1 degradation rate, light
$D_{t,d}$	0.3566	h^{-1}	TOC1 degradation rate, dark
H_c	2.5007	-	Cooperativity for *CCA1* transcription activation by TOC1
$R_{c,l}$	3.2752	$[]^{-1}$	Strength of *CCA1* transcription activation by TOC1, light
$H_{c,d}$	1.3856	$[]^{-1}$	Strength of *CCA1* transcription activation by TOC1, dark
Y_c	1.3308	h^{-1}	*CCA1* mRNA degradation rate
S_c	4.9049	[]	*CCA1* transcription rate scale factor
K_c	10	h^{-1}	Rate of CCA1 transport to nucleus
$D_{c,l}$	0.4242	h^{-1}	CCA1 degradation rate, light
$D_{c,d}$	0.2694	h^{-1}	CCA1 degradation rate, dark
D_u	0.1829	h^{-1}	Luciferase degradation+deactivation rate
Y_u	1	h^{-1}	Luciferase mRNA degradation rate
