# Supplementary material for: Multiple light inputs to a simple clock circuit allow complex biological rhythms
Source: Plant J. 2011 Apr;66(2):375–85. doi: 10.1111/j.1365-313X.2011.04489.x (PMC3130137; doi:10.1111/j.1365-313X.2011.04489.x)
Supplement: Supplementary file 9 [file tpj0066-0375-SD9.pdf]

Table S2. The data sets used to fit the model parameters.

ID	Light	Reporter	Data sets
LD_20080110	4*LD 12:12	all	4
LD_20060824	4*LD 12:12	all	4
LL_20070319	3*LL 24	all	4
LD_20080625_S	4*LD 8:16	all	4
LD_20080625_L	4*LD 16:8	all	4
LD_20080819_S	5*LD 8:16	all	4
LD_20080819_L	5*LD 16:8	all	4
LD_20080225_S	5*LD 8:16	all	4
LD_20080129_L	5*LD 16:8	all	4
PP_20070615_S	2*LD 12:12, 3*LD n:(24-n), 3*LL 24; $n \in \{2,4,6,8,10,12\}$	all	24
PP_20070615_L	LD 12:12, 3*LD (24-n):n, 3*LL 24; $n \in \{2,4,6,8,10,12\}$	all	24
PP_20070610_S	LL 12, 3*DL (24-n):n, LL 20; $n \in \{2,4,6,8,10,12\}$	TOC1-LUC, CCA1-LUC	12
PS_20070808_LM	LL 12, 3*DL (24-n):n, 3*LL 24; $n \in \{2,4,6,8,10,12\}$	all	24
PS_20070808_EM	DL 12:12, 3*DL n:(24-n), 3*LL 24; $n \in \{2,4,6,8,10,12\}$	all	24
