# Supplementary material for: Multiple light inputs to a simple clock circuit allow complex biological rhythms
Source: Plant J. 2011 Apr;66(2):375–85. doi: 10.1111/j.1365-313X.2011.04489.x (PMC3130137; doi:10.1111/j.1365-313X.2011.04489.x)
Supplement: Supplementary file 10 [file tpj0066-0375-SD10.pdf]

Table S3. The effects of light on the behaviour of the model at different time points.

What / when	CCA1	TOC1
Light on at dawn	Increased transcription unless the night was long.	Degradation of active form but stabilisation of inactive form, with little net effect. Most TOC1 is gone at this point anyway.
Light during the day	Increased degradation in the light causes CCA1 to drop to a low level.	In some experiments there is an unexplained minor TOC1 protein peak before noon. The inactive form starts accumulating once CCA1 is low.
Light near dusk	As the level of active TOC1 rises, so does CCA1 despite its higher degradation rate in the light.	Some conversion into the active form even in the light.
Light in long days or when dusk was expected (e.g., entry into LL)		Repression by CCA1 causes TOC1 to peak and decline. After entrainment in LD 12:12, this happens 16 hours after dawn.
Further into LL	The decline of TOC1 leads to a broad CCA1 peak in late subjective night.	With the decline of CCA1, the cycle starts anew.
Lights off at dusk	Decreased degradation and transcription cause a minor peak or bend depending on the exact state and timing.	Decreased transcription causes TOC1 to peak shortly after dusk. In long days the peak has already occurred. In short days the level is still so low at this point that the peak is not reached until further into the night.
Darkness in the night	Slow increase and peak due to active TOC1.	Activation and subsequent degradation lowers the total level throughout the night, but the active form peaks before declining.
Lights on in the subjective night	Increased transcription rate leads to a higher peak. The acute light response at dawn is strengthened but is merged with the main peak if the light is turned on more than 2–3 hours early.	The transcription rate does not recover unless there was more than 2 hours of darkness, but the protein level rises for several hours before dropping again. This is unexplained in the model but might depend on long-lived mRNA that is degraded upon translation.
