# Supplementary material for: Multiple light inputs to a simple clock circuit allow complex biological rhythms
Source: Plant J. 2011 Apr;66(2):375–85. doi: 10.1111/j.1365-313X.2011.04489.x (PMC3130137; doi:10.1111/j.1365-313X.2011.04489.x)
Supplement: Supplementary file 11 [file tpj0066-0375-SD11.pdf]

**Appendix S1:** Modelling details

*Model equations*

The full core (non-reporter-specific) equations are:

$$\frac{da}{dt}=A(ql-a)$$

$$\frac{dt_m}{dt}=O_t+\frac{L_t+R_a a}{1+L_t+R_a a+(R_c E_c c_N)^{H_t}}-Y_t t_m$$

$$\frac{dt_I}{dt}=S_t t_m-\left(l K_{t,l}+(1-l)K_{t,d}\right)t_I$$

$$\frac{dt_A}{dt}=\left(l K_{t,l}+(1-l)K_{t,d}\right)t_I-\left(l D_{t,l}+(1-l)D_{t,d}\right)t_A$$

$$\frac{dc_m}{dt}=O_c+\frac{\left(\left(l R_{c,l}+(1-l)R_{c,d}\right)E_t t_A\right)^{H_c}}{1+\left(\left(l R_{c,l}+(1-l)R_{c,d}\right)E_t t_A\right)^{H_c}}-Y_c c_m$$

$$\frac{dc_C}{dt}=S_c c_m-K_c c_C-\left(l D_{c,l}+(1-l)D_{c,d}\right)c_C$$

$$\frac{dc_N}{dt}=K_c c_C-\left(l D_{c,l}+(1-l)D_{c,d}\right)c_N$$

The parameters $O_t$ and $O_c$ can be made non-zero to simulate overexpression of either gene. The parameter $E_c=1$, except that for the CCA1-LUC reporter, $E_c>1$ if the CCA1-LUC fusion protein is active in regulation. The same applies to $E_t$ for TOC1-LUC. To simulate experiments with transcriptional reporters we add variables for LUC and its mRNA. For pTOC1::LUC the extra equations are:

$$\frac{du_m}{dt}=\frac{L_t+R_a a}{1+L_t+R_a a+(R_c c_N)^{H_t}}-Y_u u_m$$

$$\frac{du}{dt}=u_m-D_u u$$

For pCCA1::LUC they are:

$$\frac{du_m}{dt}=O_c+\frac{\left(\left(l R_{c,l}+(1-l)R_{c,d}\right)t_A\right)^{H_c}}{1+\left(\left(l R_{c,l}+(1-l)R_{c,d}\right)t_A\right)^{H_c}}-Y_u u_m$$

$$\frac{du}{dt}=u_m-D_u u$$

For experiments with translational reporters, we instead add variables for the luciferase-fused mRNA and the active luciferase-fused protein species. For TOC1-LUC:

$$\frac{dt'_m}{dt}=\frac{L_t+R_a a}{1+L_t+R_a a+(R_c c_N)^{H_t}}-Y_t t'_m$$

$$\frac{dt'_I}{dt}=S_t t'_m-\left(l K_{t,l}+(1-l)K_{t,d}+D_u\right)t'_I$$

$$\frac{dt'_A}{dt}=\left(l K_{t,l}+(1-l)K_{t,d}\right)t'_I-\left(l D_{t,l}+(1-l)D_{t,d}+D_u\right)t'_A$$

For CCA1-LUC:

$$\frac{dc'_m}{dt} = O_c + \frac{\left(\left(l R_{c,l} + (1-l) R_{c,d}\right) t_A\right)^{H_c}}{1 + \left(\left(l R_{c,l} + (1-l) R_{c,d}\right) t_A\right)^{H_c}} - Y_c c'_m$$

$$\frac{dc'_C}{dt} = S_c c'_m - K_c c'_C - \left(l D_{c,l} + (1-l) D_{c,d} + D_u\right) c'_C$$

$$\frac{dc'_N}{dt} = K_c c'_C - \left(l D_{c,l} + (1-l) D_{c,d} + D_u\right) c'_N$$

We have assumed that the deactivation rate of luciferase in TOC1-LUC and CCA1-LUC equals its total rate of deactivation and degradation in the transcriptional lines, $D_u$, because luciferase is only subject to slow non-specific protein degradation.

*Model cost function*

For the transcriptional reporters, the observable to be fitted is $u$, whereas for the translational reporters it is the sum over the two forms or compartments, $t'_I + t'_A$ or $c'_C + c'_N$. Consider a single data set of $N$ points. Let $\bar{y}$ be the measured time series and $\bar{y}'$ the simulated one, with measurements taken at timepoints $\bar{t}$. Find for each timepoint $i$ the window of $N \cdot \min\left(1, (t_{end} - t_0)/72\right)$ points that is centered (as far as possible) on $t_i$. For all points $j, j'$ in that window, calculate the weight matrix

$$W_{i,j} = \frac{w_i(t_i - t_j)\, y_j}{\sum_{j'} w_i(t_i - t_{j'})\, y_{j'}^{\,2}},$$

where the weights are

$$w_i(\Delta t) = \left(1 - \left(\frac{|\Delta t|}{r_i}\right)^3\right)^3,$$

where $r_i$ is the greatest $|\Delta t|$ within the window.

The cost for a single data set is

$$E_e = \frac{\sum_i \left(\frac{y'_i}{\overline{W}_i \cdot \bar{y}'} - y_i\right)^2}{N \sum_i \left(\|\overline{W}_i\|^{-1} - y_i\right)^2},$$

where the denominator normalizes the cost so that a value of 1 means “as good as a straight line”.

The total cost is

$$E = \sqrt{\frac{\sum_e z_e E_e}{\sum_e z_e}},$$

where $z_e$ is the weight of each data set. This weight is set to 3 for LL data and 1 for all other data sets (see Table S2).
