# Supplementary material for: Multiple light inputs to a simple clock circuit allow complex biological rhythms
Source: Plant J. 2011 Apr;66(2):375–85. doi: 10.1111/j.1365-313X.2011.04489.x (PMC3130137; doi:10.1111/j.1365-313X.2011.04489.x)
Supplement: Supplementary file 12 [file tpj0066-0375-SD12.doc]

**Appendix S2: Damping oscillations in LL**

Cells that were released into constant light (LL) after entrainment in LD showed damped oscillations, usually observed to tend towards the trough level of the luminescence curve. Damping of the signal might be caused by loss of synchrony between cells, in which case the signal level would exponentially approach its mean, or by damping of the clocks in the individual cells, in which case we might expect an exponential decay to any level. A third possibility is a decrease in signal, due to experimental factors that affect cell health or luciferin concentration, in which case the signal will decay towards zero not only in LL but also in LD, at a rate that might vary among experiments.

We observed a relatively slow, approximately exponential decay of the signal towards zero in many of the experiments. The time scale for this decay is days to weeks. We have chosen to correct for this effect in most of the figures. Figure S5b shows how the signal decay rate differed between two LL time courses, suggesting that it might be experimentally-derived.

Correcting for this experiment-specific signal decay allowed the damping of the clock to be quantified. The oscillations in LL were found to damp to their mean level with a time constant of just 18 h, in both time courses. We have not yet been able to image the rhythms of individual cells, so it is impossible to distinguish whether the clocks damp towards the mean in single cells, or merely lose synchrony among the population. However, the relatively rapid damping of the population-level signal seems difficult to explain through desynchrony alone. We therefore suggest that the clocks of individual cells are damping under constant light, as the model also does, though we cannot measure the quantitative balance between damping and desynchrony.
